# Phosphorus-Use Efficiency Modified by Complementary Effects of P Supply Intensity With Limited Root Growth Space

**DOI:** 10.3389/fpls.2021.728527

**Published:** 2021-09-27

**Authors:** Haiqing Gong, Bilisuma Kabeto Wako, Yue Xiang, Xiaoqiang Jiao

**Affiliations:** Department of Plant Nutrition, Key Laboratory of Plant-Soil Interactions, Ministry of Education, National Academy of Agriculture Green Development, China Agricultural University, Beijing, China

**Keywords:** AM colonization, maize, phosphorus supply intensity, space availability, trade-off

## Abstract

Space availability and the maintenance of adequate phosphorus (P) supply in the root zone are essential for achieving high yield and P-use efficiency in maize production by manipulating the root morphology and arbuscular mycorrhizal (AM) fungi colonization. A major trade-off exists between root growth and AM colonization that is influenced by soil P supply intensity and space availability. However, how soil P manipulates the root morphological characteristics and AM colonization to compensate for the limitation of root-growth space induced by high-planting density is not clear. Therefore, pot experiments were conducted to investigate interactions between the root growth and AM fungi by optimizing soil P supply to compensate for limited root growth space induced by high-planting density. Similar shoot biomass and P uptake values were obtained in P200 (200 mg P kg^−1^ soil) under D = 40 (i.e., diameter of the pot is 40 cm) and P400 under D = 30, and similar values were obtained for root length, tap root length, root angle, lateral root density, and AM colonization. However, the improvement in P supply in the root zone, shoot biomass, and P uptake in P400 under D = 20 were lower than in P200 under D = 30, and there were no significant differences in the root parameters between P200 and P400 under D = 20; similarly, the root growth and AM colonization exhibited similar trends. These results suggest that optimizing P supply in the root zone to regulate the interaction between root morphological traits and AM colonization can compensate for limited root-growth space. Although P supply in the root zone increased after the root-growth space was compressed, it could not meet the P demand of maize; thus, to achieve the most efficient use of P under intensive high-density maize production, it is necessary to optimally coordinate root growth space and P supply in the root zone.

## Introduction

The sustainable use of phosphorus (P) is a major challenge in agricultural production, especially in high-density planting systems (Testa et al., 2016). High planting density reduces the growth space of maize roots per plant; such conditions are often accompanied by smaller roots per plant, reduced soil volume occupied by the root system, and decreased the total root length (Wang et al., 2020). Maize plants primarily depend on the root morphological traits to enhance P acquisition in the soil (Lambers et al., 2015; Wang et al., 2020). The low root growth spaces are induced by high planting density, which results in difficulty in acquiring P from the soil (Shao et al., 2018). Artificially increasing P supply is an effective method of regulating the P absorption capacity of plants to meet the P demand of high-density populations (Poorter et al., 2012). In intensive agriculture, smallholders often employ the insurance approach, that is, large amounts of mineral P fertilizer are applied in excess to increase the P availability to crop plants, resulting in low P-use efficiency (PUE) (Dhillon et al., 2017; Zhang et al., 2019), with considerable adverse environmental impacts, such as non-point source pollution of surface waters (Ni et al., 2015). Therefore, both the P supply and root growth space availability should be considered when attempting to increase the PUE under intensive high-density planting maize systems.

The P-supply intensity and root-growth-space availability often affect the root morphology and arbuscular mycorrhizal (AM) colonization (Zhang et al., 2017; Shao et al., 2018; Wang et al., 2020), and in turn, impact PUE under maize production. Lyu et al. (2016) showed that at the root soil interface, available P could be enhanced by altering the root morphology. Moreover, P has been shown to stimulate root development and subsequent P uptake (Ma et al., 2020). In addition, P availability can profoundly influence the plant responses to AM colonization (Campos et al., 2018). The high level of P can inhibit AM colonization, reducing P uptake, and the benefits of AM colonization (Breuillin et al., 2010). Furthermore, increasing evidence demonstrates that high plant density can affect the plant root morphology and AM colonization (Schroeder and Janos, 2004; Niu et al., 2020). Space availability decreases with the increasing plant density, and the root growth is subsequently inhibited. In maize, the high plant density reduces the root to shoot ratio, root biomass, and root length (Shao et al., 2018); however, root length density increases with high-density planting, which increases the chances of roots crossing and overlapping, and could easily lead to P deficiency in the rhizosphere (Postma et al., 2014; Li et al., 2019). In contrast, AM colonization increases with an increase in the planting density, as AM fungi can partially alleviate P deficiency stress (Abdel-Fattah and Asrar, 2012). Hence, a trade-off exists between the root morphological traits and AM colonization to enhance P acquisition under intensive P supply and space availability; however, the mechanism of interactive effects of root morphological traits and AM colonization on maize P uptake is not fully understood. A deeper understanding of the mechanisms of root and AM interactive effects on the root growth space exploitation and maize P uptake is key to fostering high PUE.

The high plasticity of root system architecture and AM fungi responses to P supply intensity and space availability have indicated a high potential to improve PUE in maize production (Postma et al., 2014; Li et al., 2019; Shao et al., 2019). For example, PUE in maize production can be improved by manipulating the root morphology and AM colonization to maintain an appropriate P supply intensity in the root zone (Deng et al., 2017; Kobae, 2019; Chu et al., 2020). Similarly, maintaining space for proper root growth in intensive high-plant-density maize production systems could modify the root morphology and AM colonization to enhance maize yield (Yu et al., 2019; Hou et al., 2021). However, most of the previous studies in high-density planting systems have focused on the regulation of root morphology. Our understanding of how AM colonization in high-density planting systems responds to P supply is poor (Ma et al., 2013; Teng et al., 2013; Testa et al., 2016), in addition to how altered P supply affects the root growth and AM colonization to compensate for limited root growth space. To simultaneously achieve high maize yield and PUE in high-density planting systems, it is necessary to comprehensively consider P supply in the root zone and root-growth space by modifying root morphology and AM colonization. An improved understanding of the compensatory effects of maize root expansion on the root growth space, under P supply intensity regulation, would further enhance our understanding of the biological potential for efficient P use in maize.

Here, a pot experiment was carried out with maize to determine the compensation mechanisms of soil P supply intensity on the maize root morphology, AM colonization, and P uptake under different space availability scenarios. The present study specifically aimed: (1) to investigate maize growth and P uptake under different P intensities and root-growth space treatments; and (2) to examine the compensatory effects of soil P supply on root-growth space to determine environments that enhance PUE in maize.

## Materials and Methods

### Experimental Site

A greenhouse pot experiment was conducted from June to August 2020 at the Quzhou Experimental Station of China Agricultural University (36° 51′ 57″ N, 150° 0′ 37″ E), Quzhou County, Hebei province, China. Calcareous loamy soil was collected from the Quzhou Long-Term Fertilizer Station (36° 52′ 0″ N, 115° 02′ 0″ E) of China Agricultural University, Hebei province, China. The basic soil properties were as followed: pH, 8.13; soil organic carbon (SOC), 7.3 g kg^−1^; Olsen-P, 2.40 mg kg^−1^; soil total nitrogen (TN), 0.91 g kg^−1^; and available potassium (AK), 183 mg kg^−1^. The soil was air-dried and passed through a 2-mm sieve prior to experimentation. The soil pH was measured in a 1:2.5 (w/v) soil/water slurry. SOC was determined with 0.25-mm sieved soil as explained by Cambardella et al. (2001). Soil TN was determined using the Kjeldahl method (Carter and Gregorich, 2007). Soil Olsen-P was determined in 0.5 M NaHCO_3_ extracts using Mo-Sb colorimetry (Olsen et al., 1954).

### Experimental Design and Management

The experiment consisted of a 3 × 3 complete factorial design. There were three P levels (0, 200, and 400 mg P kg^−1^ soil as CaH_2_PO_4_, hereafter referred to as P0, P200, and P400, respectively) and three pot sizes (20 cm diameter × 50 cm height, 30 cm diameter × 50 cm height, and 40 cm diameter × 50 cm height, hereafter referred to as D = 20, D = 30, and D = 40, respectively). Each of the nine treatment combinations was replicated four times to yield a total of 36 pots.

Urea and muriate of potash were applied to all the treatments at a rate of 150 kg ha^−1^ to supply nitrogen (N) and potassium (K), which are often limiting factors in the calcareous loamy soils. All fertilizers were incorporated and thoroughly mixed with the soil at the time of planting.

Maize seeds (*Zea mays* L. cv. Zhengdan958) were surface sterilized in 10% v/v H_2_O_2_ for 30 min, washed with deionized water, and placed in a dish containing aerated saturated CaSO_4_ solution at 25°C in the dark until a radicle emerged. Then, four germinated seeds of uniform size were sown in each pot. Each pot was watered daily to 80% field capacity as measured by weight. The temperature ranged from a minimum of 22°C at night to a maximum of 30°C during the day.

### Plant Harvest and Measurements

The plants were harvested 55 days after sowing. The shoots were cut at the soil surface, oven-dried at 105°C for 30 min and then, at 75°C for 3 days until they reached constant weight and weighed to record the biomass. Afterward, the shoots were crushed and homogenized. Shoot P concentration was determined using the vanadomolybdate method (Johnson and Ulrich, 1959) after wet digestion with a mixture of 5 ml of 98% H_2_SO_4_ and 8 ml of 30% H_2_O_2_.

### Soil Sampling and Measurements

Bulk soil sample was obtained 20 cm from the soil surface using a soil auger. Rhizosphere soil was obtained by brushing off the soil adhering to the whole roots with a sterile brush. Visible roots were removed manually before the samples were ground to pass through a 2-mm sieve and dried naturally. Soil Olsen-P was determined in 0.5 M NaHCO3 extracts using Mo-Sb colorimetry (Olsen et al., 1954).

### Root Sampling and Root Parameter Measurements

After cutting off the shoots, the excavated roots were shaken briefly to remove a large fraction of the soil adhering to the root crown. The root angle was assessed using the method outlined by Hecht et al. (2019). Afterward, all the visible roots in each pot were collected from the soil on a 2 mm-diameter mesh and washed clean with running water in the lab. Total root length was analyzed using Win-RHIZO software (Pro2004b, version 5.0, Regent Instruments Inc., QC, Canada). Lateral root density was the number of lateral roots per unit length of primary root, which was calculated by dividing the number of lateral roots per 5 cm of primary root by the 5 cm length of the root.

The fine roots were cut to 1 cm segments and thoroughly mixed. The root samples were cleaned with 10% (w:v) potassium hydroxide and placed in a water bath (90°C) for 2 h. The cooled root samples were cleaned in 2% HCl for 5 min and then stained with Trypan blue solution. For each sample, 15 root segments were used to evaluate AM colonization by scoring based on the findings of Trouvelot et al. (1986) and calculated using MYCOCALC program (http://www.dijon.inra.fr/mychintec/Mycocalc-prg/ download.html). AM colonization (%) = number of intersections colonized (hyphae, arbuscules, vesicles, and hyphal coils)/total number of intersections examined × 100% (Zhang et al., 2014).

All roots were collected and dried at 75°C for 72 h and weighed to determine the biomass.

### Statistical Analysis

IBM SPSS Statistics 20.0 (IBM Corp., Armonk, NY, USA) was used to compute one-way or two-way ANOVA. The mean differences among the means were determined by the least significant difference (LSD) at *P* ≤ 0.05 probability level. The data were presented as graphs prepared by Sigmaplot version 10.0 (Systat Software Inc., San Jose, CA, USA).

## Results

### Shoot Biomass and P Content

**Figure 1:**
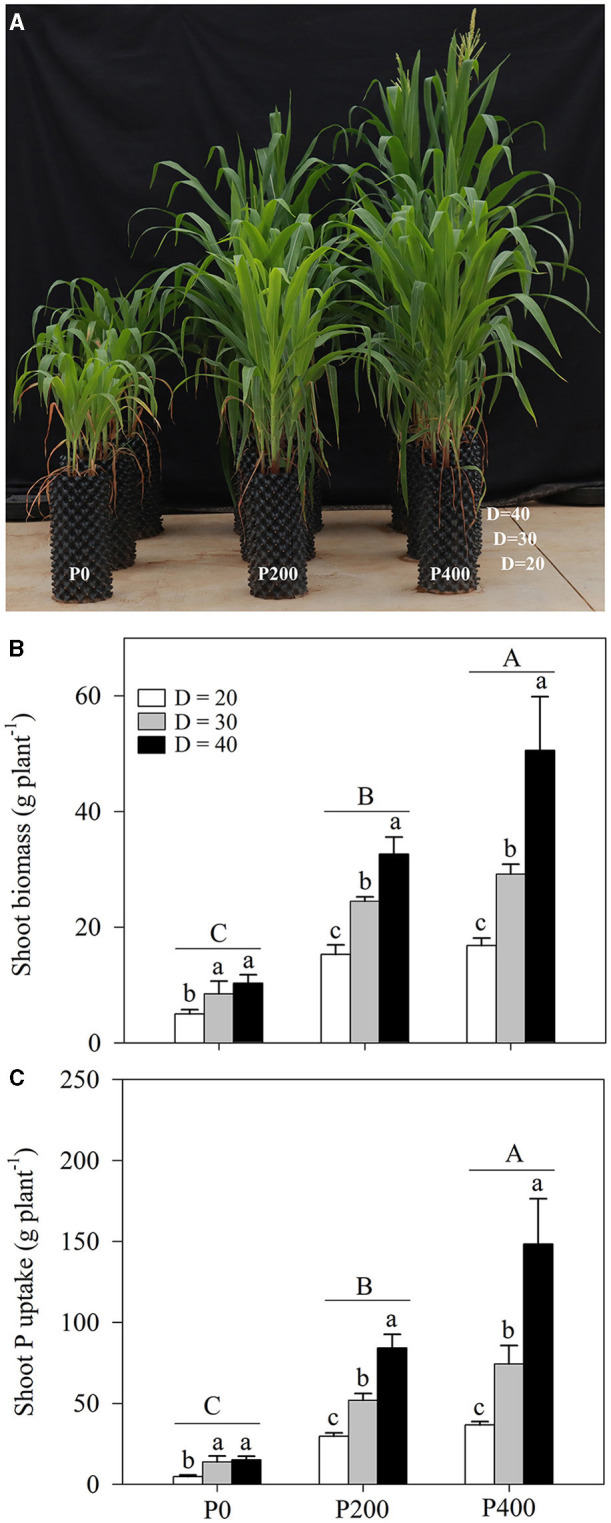
Plant growth performance **(A)**, shoot biomass **(B)**, and shoot phosphorous (P) content **(C)**, under different P supply gradients and space availability conditions. Each value represents the mean of four replicates (+SD). Different lower-case letters indicate a significant difference among different space availability levels, and different capital letters indicate a significant among different P levels. P0, 0 mg P kg^−1^ soil; P200, 200 mg P kg^−1^ soil; and P400, 400 mg P kg^−1^ soil; D = 20, D = 30, and D = 40 represent diameter of pot is 20, 30, and 40 cm, respectively.

Shoot biomass was significantly affected by soil P supply and space availability (*P* < 0.01, Table 1; Figures 1A,B; Supplementary Figures 1A,B). Shoot biomass increased as the soil P supply increased, and the highest shoot biomass was obtained in the P400 treatment groups, followed by in the P200 and P0 treatments. The effect of space availability on shoot biomass was more prominent when more P was added to the soil (P200 and P400 treatments); however, the limited space availability could restrict the growth of shoot biomass. Lower shoot biomass was obtained with the D = 20 treatment among all the P supply treatments (Figures 1A,B). However, the reduction in shoot biomass due to space limitation was compensated by soil P supply with certain space availability treatments. Higher shoot biomass was obtained in P400 under D = 30, which was equivalent to the shoot biomass observed in P200 under D = 40; the shoot biomass in P200 under D = 30 was significantly higher than that in P0 under D = 40. Furthermore, the shoot biomass in P400 under D = 20 was significantly lower than in P200 under D = 30. In addition, the shoot biomass did not tend to increase under the improving P supply conditions at lower space availability treatments. Under D = 30 and D = 40, the shoot biomass significantly increased as soil P supply increased, while there was no significant difference in the shoot biomass between P200 and P400 under D = 20, indicating that the shoot biomass was restricted by the amount of space and could not be compensated by soil P supply.

**Table 1:** Two-way ANOVA of the effects of phosphorous (P) supply, space availability, and their interaction on P supply and space availability.

**Parameters**	**P supply**		**Space availability**		**P supply × Space availability**	
	***F*** **-value**	***P*** **-value**	***F*** **-value**	***P*** **-value**	***F*** **-value**	***P*** **-value**
Shoot biomass	148.9	<0.0001	87.28	<0.0001	16.17	<0.0001
Shoot P content	149.01	<0.0001	92.01	<0.0001	<0.0001	<0.0001
Root biomass	133.3	<0.0001	73.05	<0.0001	10.74	<0.0001
Root length	156.3	<0.0001	115.5	<0.0001	18.05	<0.0001
Tap root length	103.3	<0.0001	115.0	<0.0001	7.921	0.0002
Root angle	1.5712	0.2022	146.1	<0.0001	6.137	0.0012
Lateral root density	92.04	<0.0001	13.87	<0.0001	4.115	0.0099
AM colonization	1128	<0.0001	235.8	<0.0001	61.90	<0.0001
Bulk soil Olsen P	654.3	<0.0001	1.280	0.0701	1.618	0.2129
Rhizosphere soil Olsen P	1,128	<0.0001	235.8	<0.0001	61.90	<0.0001

The shoot P content in maize among the treatments was also significantly affected by soil P supply and space availability (*P* < 0.01, Table 1; Figure 1C; Supplementary Figure 1C). The shoot P content increased as soil P supply increased, and the lower rates of shoot P uptake were obtained with the D = 20 treatment across different P supply rates, when compared with D = 30 and D = 40. Similar complementary effects of soil P supply and space availability on the shoot P content were obtained under some circumstances. When compared with that in P200 under D = 20 and D = 30, higher P content was obtained under D = 40. Further, the shoot P content in P200 under D = 30 was significantly higher than in P0 under D = 40. In contrast, the shoot P content obtained in P200 under D = 30 was significantly higher than in P400 under D = 20. There was no significant difference in the shoot P content as P supply increased from P200 to P400 under D = 20 due to the space limitation.

### Root Biomass

**Figure 2:**
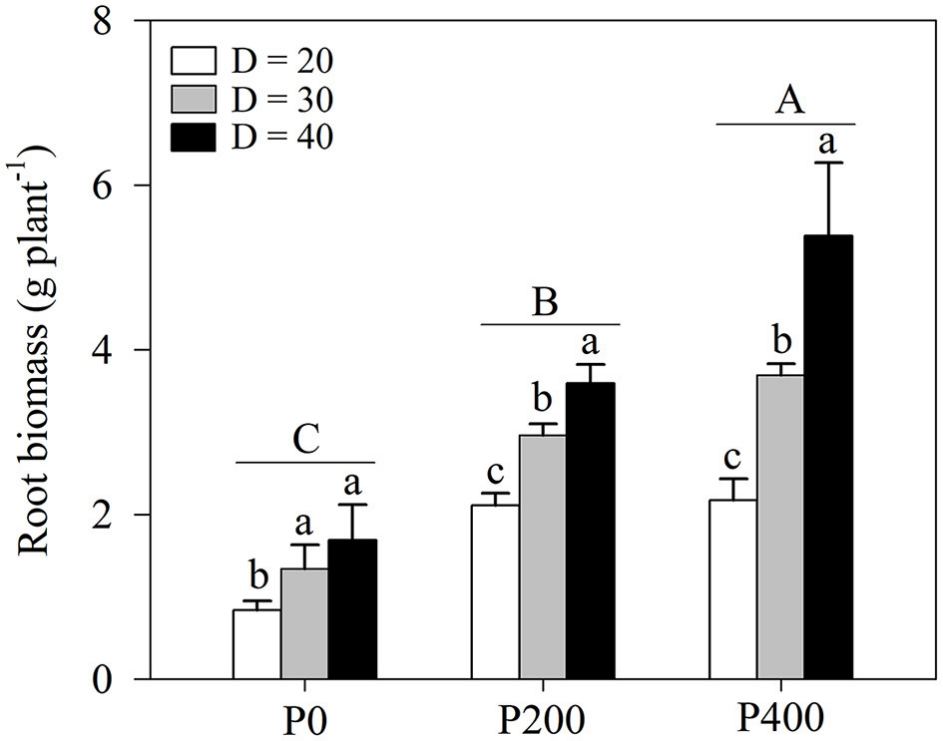
Root biomass under different phosphorous (P) supply gradients and space availability conditions. Each value represents the mean of four replicates (+SD). Different lower-case letters indicate a significant difference among different space availability levels, and different capital letters indicate a significant among different P levels (*P* < 0.05). P0, 0 mg P kg^−1^ soil; P200, 200 mg P kg^−1^ soil; and P400, 400 mg P kg^−1^ soil; D = 20, D = 30, and D = 40 represent diameter of pot is 20, 30, and 40 cm, respectively.

Root biomass was significantly influenced by soil P supply and space availability (*P* < 0.01, Table 1; Figure 2; Supplementary Figure 2). Root biomass increased as soil P supply increased, and the effect of space availability on root biomass was stronger under higher P supply (P200 and P400), while no significant difference was observed in P0 between D = 30 and D = 40. There was a strong complementary effect of soil P supply and space availability on root growth. Similar root biomass levels were observed in P200 under D = 40 and P400 under D = 30; root biomass in P200 under D = 30 was significantly higher than in P0 under D = 40. However, a higher root biomass was observed in P200 under D = 30 than in P400 under D = 20. In addition, root biomass increased as soil P supply increased under higher space availability (D = 30 and D = 40).

### Root Morphological Traits

**Figure 3:**
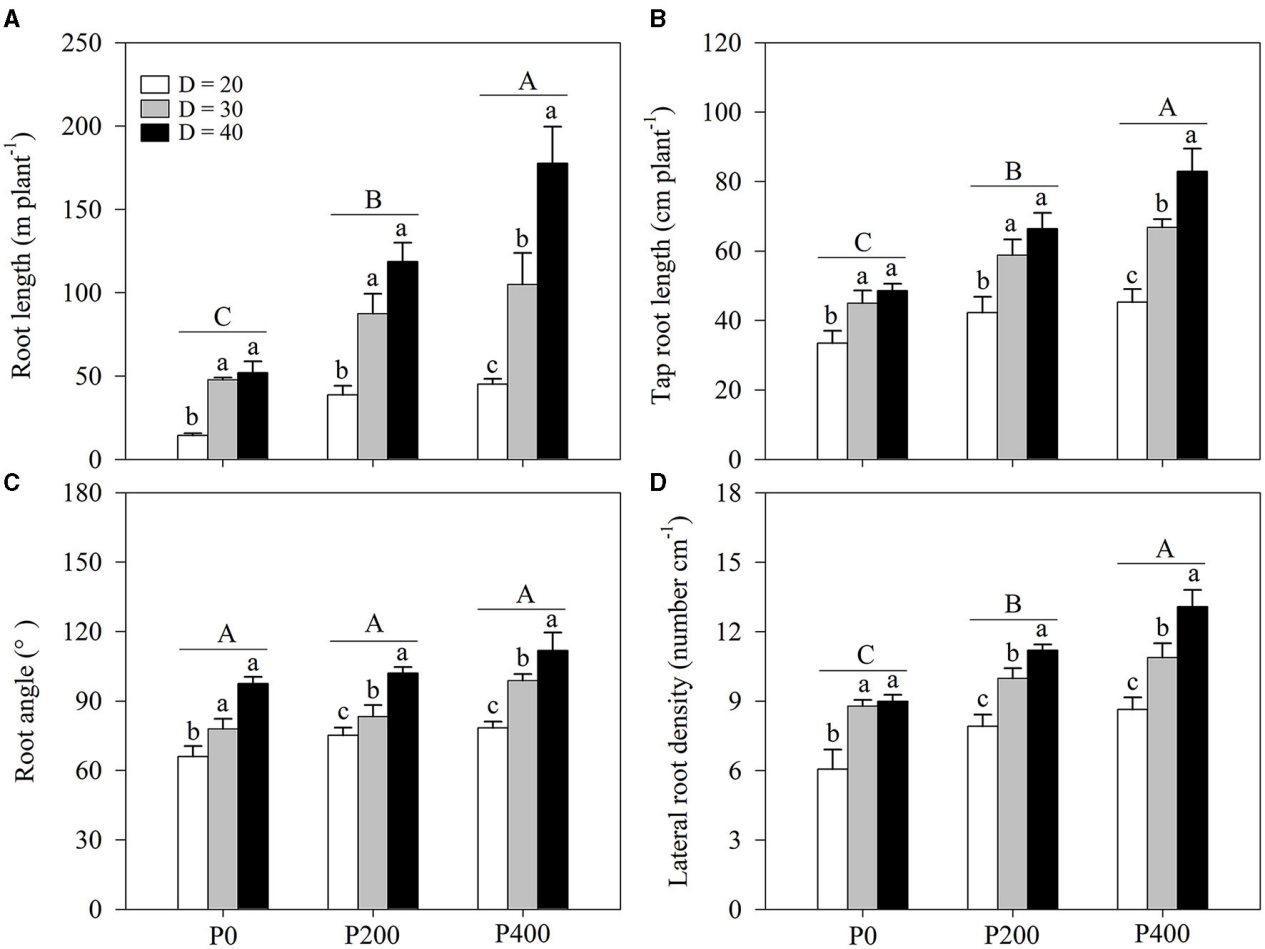
**(A)** Root length, **(B)** tap root length, **(C)** root angle, and **(D)** lateral root density under different phosphorous (P) supply gradients and space availability conditions. Each value represents the mean of four replicates (+SD). Different lower-case letters indicate a significant difference among different space availability levels, and different capital letters indicate a significant among different P levels (*P* < 0.05). P0, 0 mg P kg^−1^ soil; P200, 200 mg P kg^−1^ soil; and P400, 400 mg P kg^−1^ soil; D = 20, D = 30, and D = 40 represent diameter of pot is 20, 30, and 40 cm, respectively.

The root length was strongly influenced by soil P supply (*P* < 0.01) and space availability (*P* < 0.01) (Table 1; Figure 3A; Supplementary Figure 3A). The effect of root length increased as soil P supply increased. The effect of space availability on the root length was stronger at higher P supply levels (P400). The complementary effects of soil P supply and space availability on root length were obtained; root length obtained in P200 under D = 40 was equivalent to the root length obtained in P400 under D =30. Furthermore, the root length obtained in P200 under D = 30 was significantly higher than in P0 under D = 40; however, the root length did not increase as space availability decreased: there was no significant difference in the root length between P200 and P400 under D = 20. Finally, the root length in P400 under D = 20 was lower than that in P200 under D = 30.

Tap root length was also affected by soil P supply and space availability (Table 1; Figure 3B; Supplementary Figure 3B). Tap root length increased as space availability increased. Similar tap root length was obtained in P400 under D = 30 and P200 under D = 40 and tap root length in P200 under D = 30 was significantly higher than that in P0 under D = 40. However, there was no significant difference in tap root length between P200 and P400 under D = 20. Furthermore, tap root length in P400 under D = 20 was lower than that in P200 under D = 30.

The root angle was strongly affected by space availability, with little relationship to soil P supply (Table 1; Figure 3C; Supplementary Figure 3C). Significantly higher root angles were observed in D = 40 compared with that in D = 20 and D = 30 among all P supply treatments; however, there was no significant difference in the root angle between P400 under D = 30 and P200 under D = 40. The effect of soil P supply on the root angle was negligible under low space availability (D = 20).

Soil P supply and space availability significantly influenced the lateral root density. The lateral root density significantly increased as P supply increased, while the effect of space availability on the lateral root density was considerable when more P was added to the soil (P200 and P400). Notably, the lateral root density obtained in P400 under D = 30 was equivalent to the lateral root density in P200 under D = 40. Significantly higher lateral root density was obtained in P200 under D = 30 than in P0 under D = 40. Similar to root angle, the effect of soil P supply on lateral root density was negligible under lower space availability (D = 20). The lateral root density in P400 under D = 20 was lower than that in P200 under D = 30.

### AM Colonization

**Figure 4:**
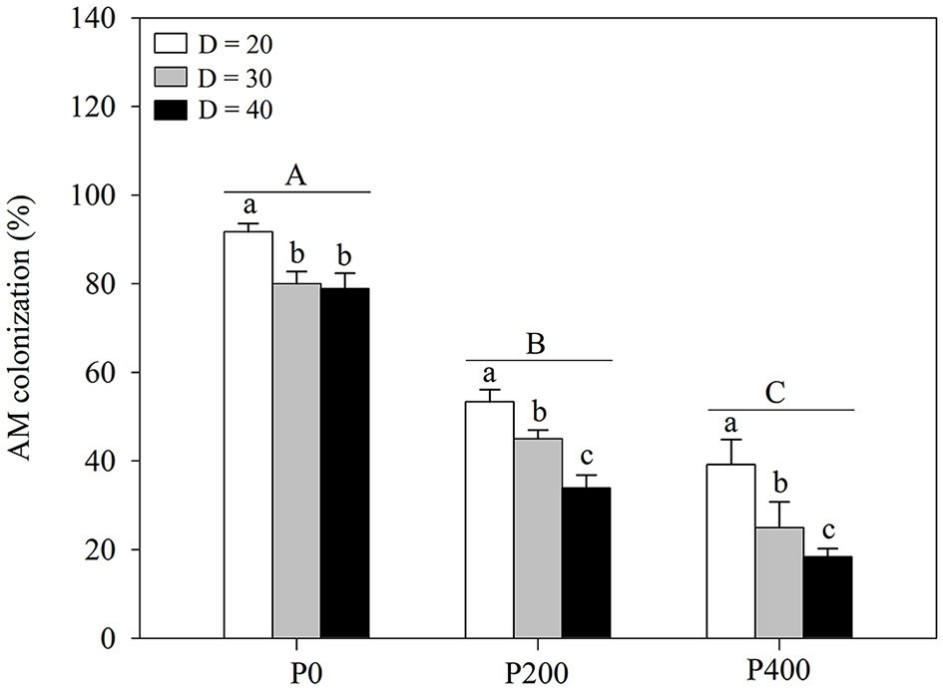
Arbuscular mycorrhizal (AM) colonization under different phosphorous (P) supply gradients and space availability conditions. Each value represents the mean of four replicates (+SD). Different lower-case letters indicate a significant difference among different space availability levels, and different capital letters indicate a significant among different P levels (*P* < 0.05). P0, 0 mg P kg^−1^ soil; P200, 200 mg P kg^−1^ soil; and P400, 400 mg P kg^−1^ soil; D = 20, D = 30, and D = 40 represent diameter of pot is 20, 30, and 40 cm, respectively.

Arbuscular mycorrhizal colonization was significantly affected by soil P supply and space availability (Table 1; Figure 4; Supplementary Figure 4); AM colonization decreased with increasing P supply from P0 to P400, regardless of space availability. Furthermore, the effect of AM colonization decreased as the space availability increased in all P treatments; P supply in the root zone and intraspecific density altered plant responses to AM colonization. Higher levels of AM colonization were observed under D = 20 compared with under D = 30 and D = 40. No significant differences were found between AM colonization in P200 under D = 40 and P400 under D = 30, while AM colonization in P0 under D = 40 was significantly higher than that in P200 under D = 30. AM colonization decreased significantly as P supply increased under D = 30 and D = 40, whereas there was no significant difference between AM colonization under conditions of higher P supply (P200 and P400) under D = 20. In addition, AM colonization decreased as space availability increased. The findings imply that the absorption of P by maize is most dependent on AM fungi at the limit of space availability, especially under P0.

### Soil Olsen-P Concentration

**Figure 5:**
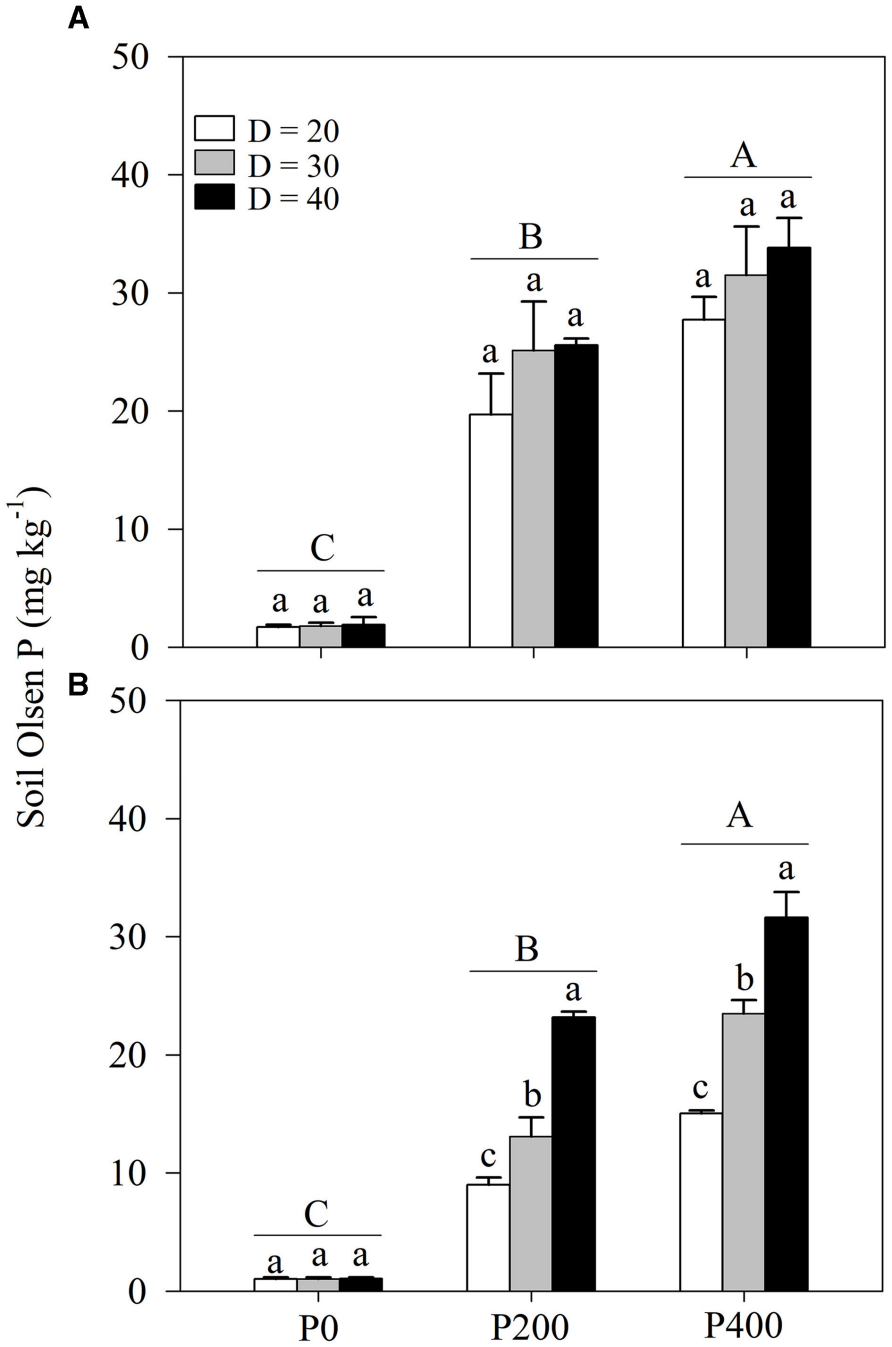
**(A)** Bulk soil Olsen phosphorous (P) and **(B)** rhizosphere soil Olsen P under different P supply gradients and space availability conditions. Each value represents the mean of four replicates (+SD). Different lower-case letters indicate a significant difference among different space availability levels, and different capital letters indicate a significant among different P levels (*P* < 0.05). P0, 0 mg P kg^−1^ soil; P200, 200 mg P kg^−1^ soil; and P400, 400 mg P kg^−1^ soil; D = 20, D = 30, and D = 40 represent diameter of pot is 20, 30, and 40 cm, respectively.

Bulk soil Olsen-P concentration was strongly affected by soil P supply, with little correlation with space availability (Table 1; Figure 5A; Supplementary Figure 5A). In contrast, the rhizosphere soil Olsen-P concentration was greatly influenced by both soil P supply and space availability (Table 1; Figure 5B; Supplementary Figure 5B). Soil Olsen-P concentration in the bulk and rhizosphere soils increased as soil P supply increased. The highest soil Olsen-P concentration was observed in P400 followed by in P200 and then P0. Higher rhizosphere soil-Olsen P concentrations were obtained in P200 under D = 40 and P400 under D = 30.

## Discussion

Plants have evolved a series of mechanisms to acquire P from the soil (Lynch, 2011). In maize, the root morphology is a major pathway for obtaining P from soils (Liu et al., 2016; Lugli et al., 2020). Therefore, in addition to P supply in the root zone, space availability for root growth is critical for optimal maize production (Lynch, 2013; Poorter et al., 2016); however, in the maize plants grown at high density, root growth space can be limited, and whether additional soil P supply can compensate for such limitation and the underlying mechanisms are not fully understood. Understanding such interactions is crucial for improving the PUE of under high maize planting density production. In the present study, similar shoot biomass and P uptake were observed in P200 under D = 40 and P400 under D = 30 (Figure 1). Furthermore, the shoot biomass and P uptake in P400 under D = 30 were significantly higher than those in P200 under D = 40. Under insufficient space for root growth, the increasing P supply in the root zone could satisfy crop P demands to a certain extent (Poorter et al., 2012). The findings suggest that the negative effects of low space availability on shoot biomass and shoot P uptake could be counteracted by the improved soil P supply for enhanced maize growth.

With reduced space availability, the photoassimilates are less frequently allocated to the roots (Poorter et al., 2012; Shah et al., 2017). The findings of the previous studies have suggested that root growth is more inhibited under high plant density than shoot growth, which may be a factor limiting nutrient uptake, especially immobilized soil P (Shao et al., 2018). In addition, under P deficiency, the plants may allocate more carbon to the belowground parts; in maize, the root to shoot ratio can increase along with the enhanced root length and lateral root density to increase the exploration of more areas (Postma and Lynch, 2011; Niu et al., 2020). Over the long-term, low P input is usually accompanied by low productivity. As P is immobilized, increasing mineral P input into the soil is a common agronomy practice used to improve productivity (Zhang et al., 2019). In the present study, both bulk and rhizosphere soil Olsen-P concentrations increased as P supply increased (Figure 5; Supplementary Figure 5), which is similar to the findings observed when alfalfa was grown in a loessial soil (Peng et al., 2020). P is a nutrient that stimulates shoot biomass production and more importantly, acts as a regulatory signal mediating changes in root architecture (López-Bucio et al., 2003; Pongrac et al., 2020). Therefore, under lower space availability, a plant requires greater P supply to build a more efficient root system with a sufficient capacity to absorb P. In the present study, root dry weight in P400 under D = 30 was similar to that in P200 under D = 40, and root dry weight in P400 under D = 30 was significantly higher than that in P200 under D = 40 (Figure 2; Supplementary Figure 2). Root growth regulation by soil P supply and space availability could explain the complementary effects of the two factors on shoot biomass and P uptake.

Changes in space availability led to the plant responses that alter the root architecture (Ramireddy et al., 2018). The reduced space availability inhibits root length, tap root elongation, and the development of lateral roots (Figures 3A,B,D; Supplementary Figures 3A,B,D). In maize, the root architecture, such as root length, root surface area, root volume, and root diameter, can decrease under high plant density (Mi et al., 2016; Shao et al., 2018). In addition, the steeper root angles were obtained in treatments with lower space availability (Figure 3C; Supplementary Figure 3C), increasing the risks of rhizosphere P deficiency (Figure 5B). However, improved AM colonization was observed with lower space availability, suggesting that the soil P acquisition predominantly relies on root-associated AM fungi under high-planting density, where the formation of a sizeable hyphal network structure in the soil benefits P uptake (Deng et al., 2017). However, the regulation of root growth through P supply intensity is closely related to crop productivity through the exploitation of the biological potential for nutrient-use efficiency enhancement (Shen et al., 2013; McNickle, 2020). Root morphology exhibits high plasticity under varying P supply levels (Wang et al., 2020); increasing P supply induces root length growth, tap root elongation, and development of lateral roots and facilitates the acquisition of P through expansion into a greater soil volume, thereby increasing the absorptive surfaces of the roots (Williamson et al., 2001). In contrast, according to the results of the present study, AM colonization decreases as P supply increased. As expected, the root length, tap root length, and lateral root density increased as soil P supply increased through increased root-soil contact, subsequently enhancing P uptake (Figures 3A,B,D; Supplementary Figures 3A,B,D), while AM colonization decreased. Notably, this study results indicated similar root length, root angle, and lateral root density values in P200 under D = 40 and in P400 under D = 30. This finding suggests that P supply intensity may compensate for the limitation of growth space through the exploitation of the biological potential of maize to enhance P uptake through the root morphological changes.

At higher planting densities, plant growth and development can be limited by soil nutrients in the root zone (Dong et al., 2015). When the nutrient content is adequate for plant growth, increases in growth space may improve productivity (Fageria and Moreira, 2011). In the present study, shoot biomass and P uptake significantly increased with an increase in space availability under P200 and P400 (Figure 1; Supplementary Figure 1). In contrast, higher P supply (P200 and P400) did not improve shoot biomass and P uptake under D = 20 (Figure 1; Supplementary Figure 1). Due to the existence of a threshold value for the efficient uptake of P by maize roots, maize cannot absorb more P than the threshold value, even if the amount of P supply is increased in a P-limited area (Santner et al., 2012). There are two plant P uptake pathways in the soil that include P uptake from soil: P uptake by roots and the AM fungal pathway (Watts-Williams et al., 2015). Root development and AM colonization are hindered under lower space availability considering that the plant density can lead to growth factor competition (Schroeder and Janos, 2004). The potential of root biology may not be fully exploited by P supply through the regulation of root morphology and AM colonization; therefore, only by matching P supply and space availability can root growth be regulated and the plant PUE can then be improved.

Collectively, the findings of the present study provide an important scientific basis for an enhanced understanding of the mechanisms *via* which soil P supply intensity could be used to counteract the adverse effects of high maize planting density on root morphology, P uptake, and PUE improvement, for sustainable crop production with high yields and high nutrient-use efficiency. Whether the advantages of soil P supply can compensate for the limited root growth spaces induced by high planting density under field conditions still requires further comprehensive studies.

## Conclusions

Both P supply in the root zone and space availability influence the PUE in maize by influencing the root morphology and AM colonization dynamics. Similar shoot biomass and P uptake were observed in the conditions with high P supply and low space availability, and in the conditions with low P supply and high space availability. The lower maize biomass observed in conditions with relatively low space availability was fully counteracted by the relatively high P supply through the modification of root morphology and AM colonization activities. Furthermore, shoot biomass and P uptake did not increase with increased P supply in the root zone after root-growth space was reduced; here, the lower maize biomass obtained under low space availability was not fully counteracted by the relatively high P supply. The findings highlight the need to optimally coordinate the root growth space and P supply in the root zone, specifically to achieve optimal PUE under the intensive high-density maize production. The results of the present study highlight that understanding the optimal P supply in the root zone and space availability are essential for high PUE under the intensive high-density maize production system.

## Data Availability Statement

The original contributions presented in the study are included in the article/Supplementary Material, further inquiries can be directed to the corresponding author/s.

## Author Contributions

XJ supervised the experiments. HG performed most of the experiments and drafted the manuscript. BK and YX contributed reagents, materials, and analysis tools. All authors discussed the results and commented on the manuscript.

## Funding

This study was supported by the National Natural Science Foundation of China (NSFC) (32172675, 31701999) and the Deutsche Forschungsgemeinschaft (DFG, German Research Foundation)-328017493/GRK 2366 (Sino-German International Research Training Group AMAIZE-P).

## Conflict of Interest

The authors declare that the research was conducted in the absence of any commercial or financial relationships that could be construed as a potential conflict of interest.

## Publisher's Note

All claims expressed in this article are solely those of the authors and do not necessarily represent those of their affiliated organizations, or those of the publisher, the editors and the reviewers. Any product that may be evaluated in this article, or claim that may be made by its manufacturer, is not guaranteed or endorsed by the publisher.
