# Supplementary material for: Phosphorus-Use Efficiency Modified by Complementary Effects of P Supply Intensity With Limited Root Growth Space
Source: Front Plant Sci. 2021 Sep 27;12:728527. doi: 10.3389/fpls.2021.728527 (PMC8503601; doi:10.3389/fpls.2021.728527)
Supplement: Supplementary file 1 [file Data_Sheet_1.docx]

Supplementary Material

# Supplementary Figures and Tables

## Supplementary Figures

**
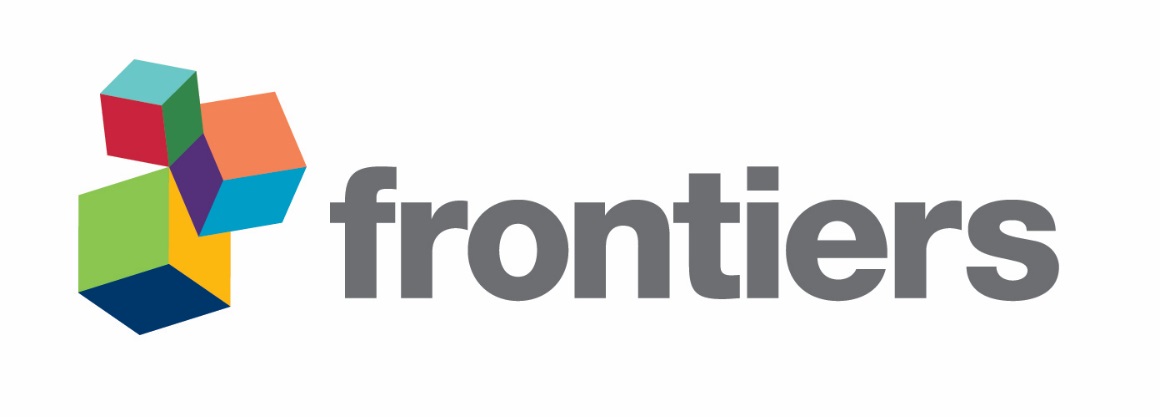
**


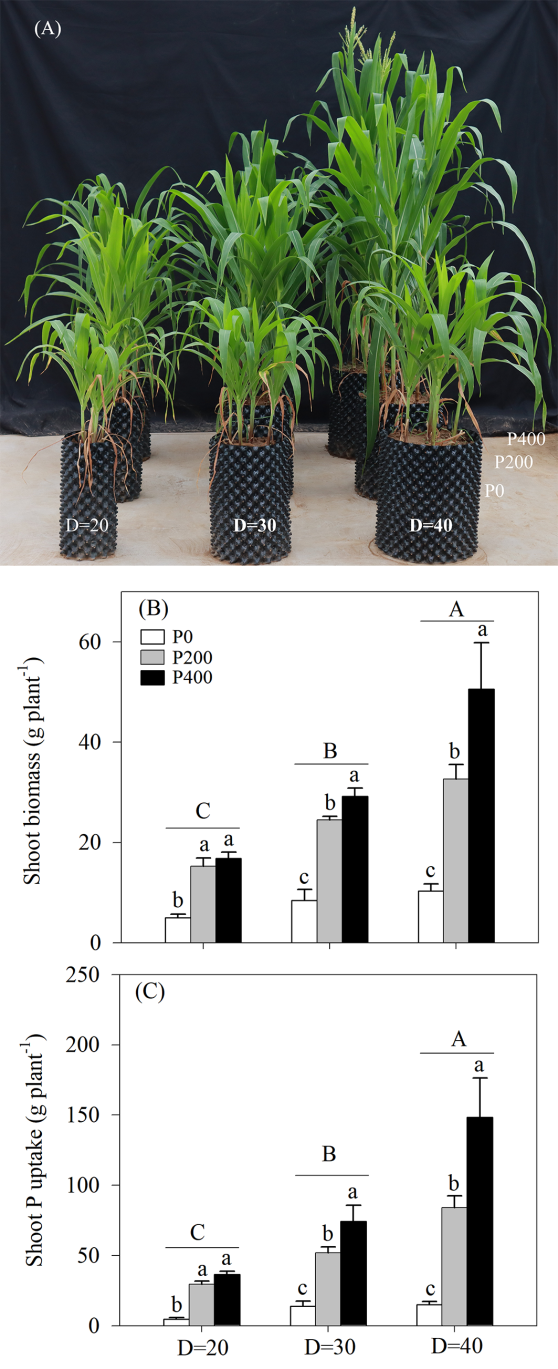


**Supplementary Figure 1.** (A) Plant growth performance, (B) shoot biomass, and (C) shoot phosphorous (P) content under different space availability conditions and P supply gradients. Each value represents the mean of four replicates (+SD). Different lower-case letters indicate a significant difference among different space availability levels, and different capital letters indicate a significant among different P levels levels (*P*<0.05). P0, 0 mg P kg^-1^ soil; P200, 200 mg P kg^-1^ soil; and P400, 400 mg P kg^-1^ soil; D=20, D=30, and D=40 represent diameter of pot is 20cm, 30cm, and 40 cm


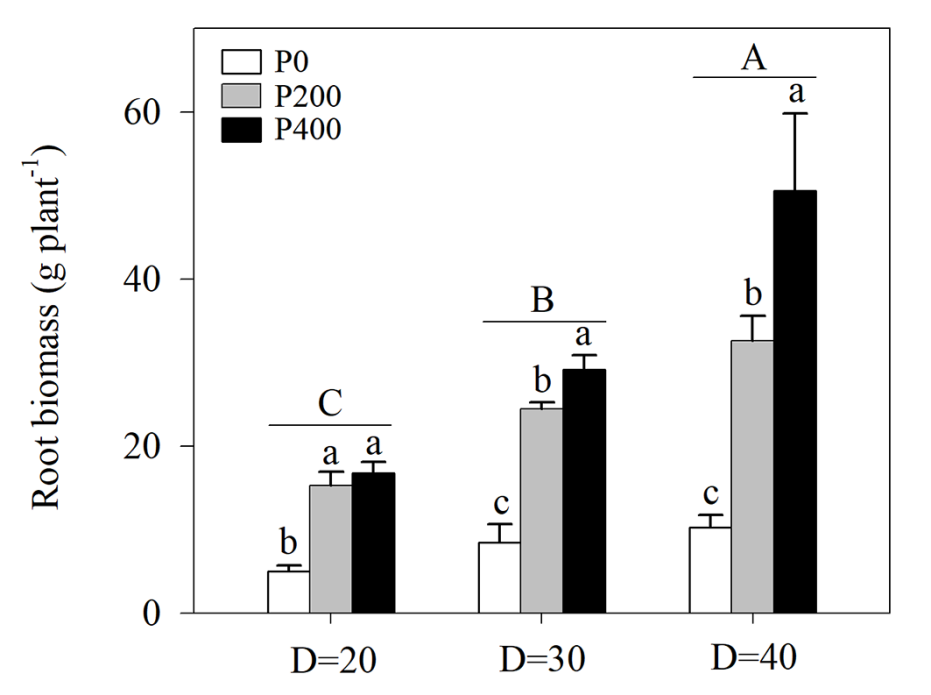


**Supplementary Figure 2.** Root biomass under different space availability conditions and P supply gradients. Each value represents the mean of four replicates (+SD). Different lower-case letters indicate a significant difference among different P levels, and different capital letters indicate a significant among different space availability levels (*P*<0.05).P0, 0 mg P kg^-1^ soil; P200, 200 mg P kg^-1^ soil; and P400, 400 mg P kg^-1^ soil; D=20, D=30, and D=40 represent diameter of pot is 20cm, 30cm, and 40 cm.


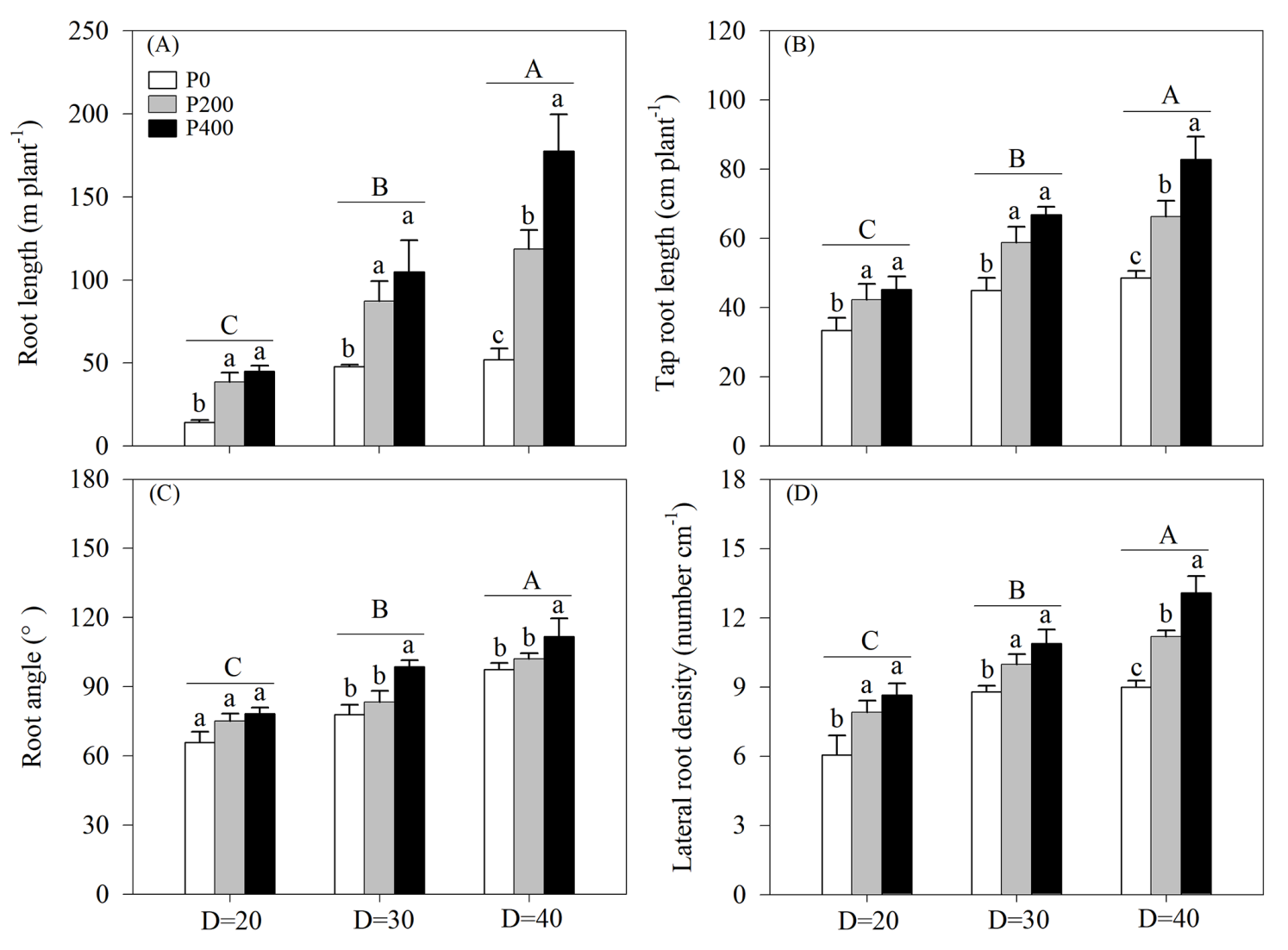


**Supplementary Figure 3.** (A) root length, (B) tap root length, (C) root angle, and (D) lateral root density under different space availability conditions and P supply gradients. Each value represents the mean of four replicates (+SD). Different lower-case letters indicate a significant difference among different P levels, and different capital letters indicate a significant among different space availability levels. P0, 0 mg P kg-1 soil; P200, 200 mg P kg-1 soil; and P400, 400 mg P kg-1 soil; D=20, D=30, and D=40 represent diameter of pot is 20cm, 30cm, and 40 cm.


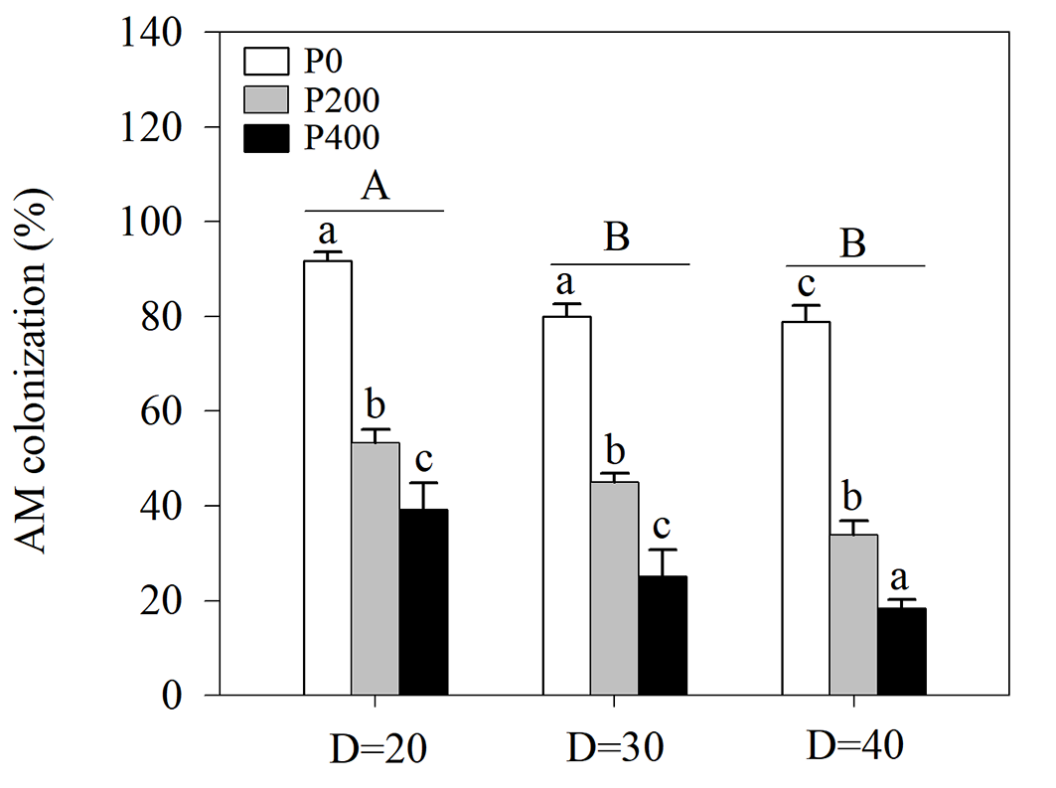


**Supplementary Figure 4.** Arbuscular mycorrhizal (AM) colonization under different space availability conditions and P supply gradients. Each value represents the mean of four replicates (+SD). Different lower-case letters indicate a significant difference among different P levels, and different capital letters indicate a significant among different space availability levels. P0, 0 mg P kg^-1^ soil; P200, 200 mg P kg^-1^ soil; and P400, 400 mg P kg^-1^ soil; D=20, D=30, and D=40 represent diameter of pot is 20cm, 30cm, and 40 cm.


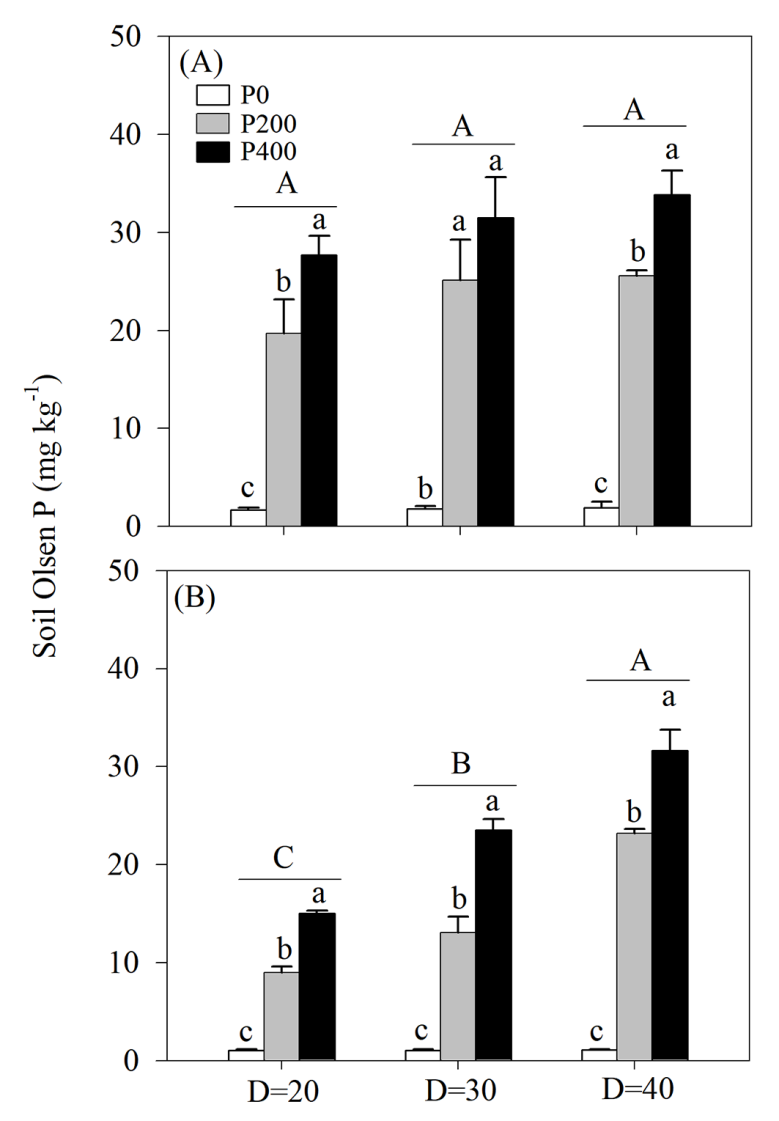


**Supplementary Figure 5.** (A) Bulk soil Olsen phosphorous (P) and (B) rhizosphere soil Olsen P under different space availability conditions and P supply gradients. Each value represents the mean of four replicates (+SD). Different lower-case letters indicate a significant difference among different P levels, and different capital letters indicate a significant among different space availability levels. P0, 0 mg P kg^-1^ soil; P200, 200 mg P kg^-1^ soil; and P400, 400 mg P kg^-1^ soil; D=20, D=30, and D=40 represent diameter of pot is 20cm, 30cm, and 40 cm.
